# Synthesis, Characterization, and In Vitro Cytotoxicity Evaluation of Doxorubicin-Loaded Magnetite Nanoparticles on Triple-Negative Breast Cancer Cell Lines

**DOI:** 10.3390/pharmaceutics15061758

**Published:** 2023-06-17

**Authors:** Jano Markhulia, Shalva Kekutia, Vladimer Mikelashvili, Liana Saneblidze, Tamar Tsertsvadze, Nino Maisuradze, Nino Leladze, Zsolt Czigány, László Almásy

**Affiliations:** 1 Nanocomposites Laboratory, Vladimer Chavchanidze Institute of Cybernetics of the Georgian Technical University, Z. Anjafaridze Str. 5, 0186 Tbilisi, Georgia; shalva.kekutia@gmail.com (S.K.); vmikelashvili@gtu.ge (V.M.); liana.saneblidze@gtu.ge (L.S.); nino.maisuradze@ens.tsu.edu.ge (N.M.); 2 Department of Biology Chair of Immunology and Microbiology, Faculty of Exact and Natural Sciences, Ivane Javakhishvili Tbilisi State University, 1, Ilia Tchavchavadze Ave., 0179 Tbilisi, Georgia; tamar.tsertsvadze@tsu.ge (T.T.); ninoleladze19@gmail.com (N.L.); 3 Institute for Technical Physics and Materials Science, Centre for Energy Research, Konkoly Thege Miklós Str. 29-33, 1121 Budapest, Hungary; czigany.zsolt@ek-cer.hu; 4 Institute for Energy Security and Environmental Safety, Centre for Energy Research, Konkoly Thege Miklós Str. 29-33, 1121 Budapest, Hungary

**Keywords:** SPIONs, triple-negative breast cancer, drug delivery, citric acid, doxorubicin

## Abstract

In this study, we investigated the cytotoxicity of doxorubicin (DOX)-loaded magnetic nanofluids on 4T1 mouse tumor epithelial cells and MDA-MB-468 human triple-negative breast cancer (TNBC) cells. Superparamagnetic iron oxide nanoparticles were synthesized using sonochemical coprecipitation by applying electrohydraulic discharge treatment (EHD) in an automated chemical reactor, modified with citric acid and loaded with DOX. The resulting magnetic nanofluids exhibited strong magnetic properties and maintained sedimentation stability in physiological pH conditions. The obtained samples were characterized using X-ray diffraction (XRD), transmission electron microscopy (TEM), Fourier-transform infrared spectroscopy, UV-spectrophotometry, dynamic light scattering (DLS), electrophoretic light scattering (ELS), vibrating sample magnetometry (VSM), and transmission electron microscopy (TEM). In vitro studies using the MTT method revealed a synergistic effect of the DOX-loaded citric-acid-modified magnetic nanoparticles on the inhibition of cancer cell growth and proliferation compared to treatment with pure DOX. The combination of the drug and magnetic nanosystem showed promising potential for targeted drug delivery, with the possibility of optimizing the dosage to reduce side-effects and enhance the cytotoxic effect on cancer cells. The nanoparticles’ cytotoxic effects were attributed to the generation of reactive oxygen species and the enhancement of DOX-induced apoptosis. The findings suggest a novel approach for enhancing the therapeutic efficacy of anticancer drugs and reducing their associated side-effects. Overall, the results demonstrate the potential of DOX-loaded citric-acid-modified magnetic nanoparticles as a promising strategy in tumor therapy, and provide insights into their synergistic effects.

## 1. Introduction

Cancer is a large group of diseases in which genetic changes disrupt the orderly division and growth of specific cells in the body, and thus this process becomes continually unregulated and leads to the malignant proliferation of cancer cells. In this group, breast cancer is the most commonly diagnosed invasive tumor formation in women worldwide and is also the leading cause of female cancer mortality [1,2,3,4,5].

Breast cancer is a heterogeneous disease that differs among different patients and even within each individual tumor [6,7]. This disease encompasses diverse subtypes, including invasive ductal carcinoma, invasive lobular carcinoma, and TNBC, each characterized by distinct biological features and treatment challenges [5,8,9,10].

Triple-negative breast cancer (TNBC) is an uncommon subtype of breast cancer, lacking expressions of all three of the aforementioned receptors [10,11]. TNBC accounts for approximately 10–15 percent of the total number of breast cancer cases diagnosed in women [12,13]. Histologically, these cancers show high malignancy and aggressive tumor biology and are associated with a poor prognosis and a high relapse rate [10,13,14].

In general, the early stages of breast cancer are treated with a combination of surgery, local irradiation, and, in some cases, adjuvant chemotherapy or hormonal therapy. Preoperative chemotherapy is also possible when the tumor size is small or surgery is difficult to perform, and this combination provides an excellent long-term prognosis [15,16].

TNBC remains a challenge despite significant efforts in traditional (standard) therapies, including definitive surgery [17,18]. Due to the heterogeneity of the disease and the lack of an identified molecular target, the therapeutic options for TNBC are limited [9]. Nevertheless, conventional chemotherapy is still the major therapeutic approach for this subgroup of patients [14,15]. It is also noteworthy that this subtype of tumor exhibits especially chemoresistant phenotypes, recurrence, and metastases, which further complicate the treatment [19,20].

Based on many scientific experiences and observations of patients in cancer treatment, such disadvantages as drug resistance and serious side-effects can accompany the use of just a single chemotherapeutic agent [21,22,23]. Therefore, there is an urgent need to develop new effective therapeutic strategies and integrated approaches that will solve critical issues in cancer treatment [6,24].

Recently, the integration of tremendous advances in nanotechnology and oncology has shown promising prospects and resources for various medical applications, leading to new, customized approaches to therapeutic drug delivery [24,25]. Multifunctional nanoparticle systems with built-in targeted drug delivery and efficient diagnostic capabilities, known as theranostic agents, hold great promise for significant improvements in biological detection and cancer treatment using a single therapeutic platform [25,26,27,28]. Among new cancer treatment strategies, exosomes also deserve attention as new platforms for drug delivery and diagnostic applications [5,29].

Because of important characteristics, such as tunable size-dependent magnetic properties, iron oxide nanoparticles (IONPs), specifically magnetite (Fe_3_O_4_) or maghemite (γ-Fe_2_O_3_), have found widespread application in cancer theranostics [26,30,31]. Compared to conventional chemotherapeutic agents, therapeutic nanoparticles tend to efficiently deliver the chemotherapeutic drug to the pathological site while avoiding toxicity in healthy organs and tissues through convection and diffusion, known as the enhanced permeability and retention (EPR) effect and active cellular uptake [1,32]. Although the scientific literature reports potential limitations of the EPR effect in clinical trials, this effect is believed to vary among individuals in the human population [1,33].

In addition, magnetic nanoparticles have another remarkable property—internal enzymatic activity [34,35]. For example, iron oxide magnetic nanoparticles have demonstrated an ability to produce reactive oxygen species (ROS) [36,37] and can also initiate a new iron-dependent form of programmed cell death—ferroptosis [37,38]. The discussion of this phenomenon is presented in a recent review [38], where the generation of ROS by doxorubicin-loaded iron oxide nanoparticles and their effect on bacteria and cancer cells is discussed. IONPs with doxorubicin were shown to induce oxidative stress and, owing to their superparamagnetic characteristic, can be prospective for magnetic-field-mediated anticancer therapy [39,40].

Fe_3_O_4_ magnetic nanoparticles were found to exhibit pH-dependent intrinsic peroxidase-like and catalase-like activities. Nanoparticles with similar properties are referred to as enzyme-mimetic nanoparticles-nanozymes [34,41]. Compared to natural enzymes, nanozymes have unique advantages, including high catalytic activity and stability under harsh conditions (pH and temperature), relatively low cost of production, and easy scalability [34,35].

The foregoing highlights once again the promising prospects for the use of magnetic nanoparticles in reactive oxygen species (ROS)-mediated anticancer therapy, which demonstrates the benefits of chemodynamic therapy (CDT) due to their high efficiency and less toxic side-effects [42,43,44,45].

It should be mentioned that magnetic nanoparticles utilized in biomedical applications need to possess particular, complicated characteristics. Surfactant molecules coating the nanoparticles can provide a new platform for further modification, broadening the scope of such nanosystems into biomedical applications while also providing colloidal stability and biocompatibility [46,47].

Among many small organic molecules, citric acid (CA, C_6_H_8_O_7_), with three carboxyls (COOH) and one hydroxyl (OH) group, is a highly water-soluble compound with strong polarity, so it can be used as a surfactant to obtain stable magnetic water-based nanoparticles [48,49]. It can be chemisorbed on the surface of the magnetite nanoparticles via one of the two carboxylate groups. Depending on the pH of the medium, the carboxyl group with one or two negative charges of citric acid remains free, which in turn can be used for modification (loading of drugs, conjugation of biomolecules, etc.) [50].

Anthracyclines such as doxorubicin (DOX) are advanced chemotherapeutic agents with a broad spectrum of antitumor activity. It is one of the most promising drugs for the successful therapy of metastatic breast tumors. However, doxorubicin-based chemotherapy is characterized by unfavorable cell selection. As a result, free anthracyclines can be found in both tumors and normal tissues and cause harmful side-effects [30,51]. Numerous efforts have been made to diminish the necessary dosage and side-effects by developing suitable drug carriers, among which the iron oxide nanoparticles are one of the favorite choices [52,53,54].

The aim of the present research was the synthesis of iron oxide magnetic nanoparticles by a modernized method of chemical coprecipitation, modification of the obtained nanoparticles with biocompatible organic molecules, and further conjugation with an antitumor drug doxorubicin. Further, determination of the physicochemical characteristics of the synthesized samples was performed, as well as the evaluation of the effect of various combinations and concentrations of the obtained magnetic nanosystem on the cytotoxicity and viability of human and mouse breast cancer cell cultures (MDA-MB-468 and 4T1) in in vitro models.

We present results obtained on a newly developed formulation of citric-acid-coated superparamagnetic iron oxide nanoparticles loaded with doxorubicin, employing electrohydraulic discharge treatment of the nanoparticles. This formulation offers enhanced stability, drug loading capacity, and the possibility for targeted drug delivery to cancer cells. Further, the results of viability studies of the investigated cells indicate a synergistic effect on cytotoxicity of the IONPs in combination with DOX on breast cancer cells, providing valuable insights into the potential synergistic effects of magnetic nanoparticles and chemotherapy drugs for cancer therapy, paving the way for further advancements in the field.

## 2. Materials and Methods

### 2.1. Reagents

The following analytical reagents were used without additional purification: iron(III) chloride hexahydrate (FeCl_3_·6H_2_O) (≥98%, Sigma-Aldrich, Darmstadt, Germany), iron(II) sulfate heptahydrate (FeSO_4_∙7H_2_O) (≥99%, Carl Roth Gmbh + Co. KG, Karlsruhe, Germany), ammonium hydroxide (NH_4_OH) solution (≥25%, Carl Roth Gmbh + Co. KG, Karlsruhe, Germany), citric acid monohydrate (ACS reagent, ≥99.0% Sigma-Aldrich, Darmstadt, Germany), doxorubicin (Doxorubicin Kocak Farma, Istanbul, Turkey), and sodium chloride 0.9 *w*/*v* (Intravenous Infusion) (B. Braun, Melsungen, Germany). Deionized water (DW) was used as a solvent.

### 2.2. Synthesis of Magnetic Nanoparticles

The synthesis of a nanofluid containing magnetic iron oxide nanoparticles was carried out using coprecipitation in an automated reactor. Some samples were additionally subjected to an electrohydraulic discharge (EHD) treatment at one stage of the synthesis.

The synthesis was carried out by modifying the standard procedure for chemical coprecipitation, namely, the controlled coprecipitation was performed in an inert gas (N_2_) atmosphere in a sonochemical reactor connected to the automated technology line.

*Stage I (synthesis)* Before carrying out the reaction in the sonochemical reactor, the deionized water was degassed using a Schlenk line vacuum system, then powders of salts of two and trivalent iron FeSO_4_∙7H_2_O, 17 mM (4.89 g); FeCl_3_∙6H_2_O, 33 mM (9.0 g) (solution molarity 0.1 M) were added to the degassed water.

The dissolution of iron salts was carried out in an atmosphere of inert gas (nitrogen) at 45 °C for 15 min using ultrasonic treatment (60% of a 900 W power homogenizer), after which the temperature was increased to 55 °C and a 14 mM aqueous solution of ammonium hydroxide was added (within 30 min) through peristaltic pumps. Then, the obtained magnetic colloid was cooled to room temperature, under ultrasonic treatment (power 30%).

*Stage II (decantation)* At this stage, the obtained magnetic colloid containing iron oxide nanoparticles was washed away from the undesirable products (ammonium chloride and sulfate) formed by the coprecipitation reaction by decantation onto a permanent magnet until pH = 6.5. After washing, 500 mL of DW was added, resulting in the concentration of magnetite in the solution equal to 7.7 mg/mL.

*Stage III (electrohydraulic and ultrasonic processing)* An amount of 300 mL from the final fluid was divided into three parts, 100 mL each. The first part contained bare iron oxide nanoparticles (BIONs), and these samples are referred to as S0. From the remaining two parts, one was processed with an electrohydraulic discharge in vacuum for 15 min, and the other part was treated in an ultrasonic reactor under vacuum for 30 min at a power of 300 W).

*Stage IV (modification with CA and DOX-loading)* Magnetic colloids, treated with ultrasonication and electrohydraulic discharge, were separately modified with citric acid molecules in an ultrasonic reactor. A previously prepared aqueous solution of citric acid (0.19 g of CA + 10 mL DW) was dropwise-added to the magnetic fluid, using a peristaltic pump at 60 °C for 30 min (processing power 270 watts, mode 2 on, 1 off). These samples were denoted as CA-SPIONs, or briefly S1, and CA-SPIONs-EHD, or briefly S2.

Doxorubicin-loaded variants of samples S1 and S2, referred to as DOX-CA-SPIONs and DOX-CA-SPIONs-EHD (or S3 and S4, respectively), were prepared separately. For the in vitro studies, samples S3 and S4 were provided in three different ways: S3 (1:1), S3 (1:5), and S3 (1:10); S4 (1:1), S4 (1:5), and S4 (1:10). These ratios indicate the mass ratio of the anticancer drug DOX to CA-SPIONs (see Section 3.8.1). The doxorubicin loading process involved adding the appropriate amount of the drug to nanosuspensions (S1, S2) and gently shaking them for 15 min at room temperature.

### 2.3. Samples’ Preparation for Doxorubicin Loading Study

Doxorubicin adsorption (loading) kinetics was evaluated for 0.1% DOX-CA-SPIONs 1:5 and DOX-CA-SPIONs 1:10 for two values of pH 4.5 and 7.2. An amount of 12.66 mL of 0.1% (*w*/*v*) CA-SPIONs aqueous nanosuspension was added to 1.18 mL of DW and 1.35 mL of 0.2% (*w*/*v*) doxorubicin hydrochloride solution. The amount of DOX in the solution (15.19 mL) was 0.166 mg/mL and that of CA-SPIONs was 0.83 mg/mL (CA-SPIONs/DOX = 5). This sample is denoted as S3–1:5. Sample S3–1:10 was prepared similarly but with twice as little doxorubicin. In more detail, in 0.64 mL of DW, we added 13.81 mL of the 0.1% (*w*/*v*) CA-SPIONs aqueous nanosuspension and 0.74 mL of 0.2% (*w*/*v*) doxorubicin hydrochloride solution (amount of DOX in entire solution was 0.09 mg/mL, that of CA-SPIONs was 0.9 mg/mL; CA-SPIONs/DOX = 10). The mixtures were gently shaken at 25 °C for 15 min. Samples to be measured (sample volume, 2.5 mL) were mixed every twelve hours for 15 min and stored in the dark in a refrigerator at a temperature of 9 °C. Measurements were made at specific times of 30 min, 15 h, 24 h, and 48 h.

### 2.4. Stability of Doxorubicin-Loaded Magnetic Nanocarriers in DW and Sodium Chloride

To study the colloidal (sedimentation) stability both in an aqueous solution and in a sodium chloride solution (NaCl), the samples were prepared in the following manner: After pre-homogenizing samples S1 and S2 of 0.04% (*w*/*v*) (0.4 mg/mL of CA-SPION in both nanofluids) for 5 min at 30% power in an ultrasonic homogenizer, 2 mL of the processed nanosuspension was extracted, to which 2 mL of deionized water and 2 mL of 0.9% (*w*/*v*) sodium chloride intravenous infusion were added. As a result, magnetic nanosuspensions S1 + DW 0.02%, S1 + NaCl 0.02%, S2 + DW 0.02%, and S2 + NaCl 0.02% were obtained. The samples S3 + DW 0.05%, S3 + NaCl 0.05%, S4 + DW 0.05%, and S4 + NaCl 0.05% of DOX-loaded nanosuspensions were prepared similarly, but without ultrasonic treatment, from 0.1% (*w*/*v*) S3 and S4 samples.

### 2.5. Cell Lines

The study was conducted using two types of breast cancer cells, models MDA-MB-468 and 4T1. MDA-MB-468 breast cancer cells from the basal subgroup are a complex and aggressive tumor subtype. They lack expressions of the estrogen receptor (ER), progesterone receptor (PR), and human epidermal growth factor receptor 2 (HER2), which makes them difficult to target therapeutically.

4T1 is a breast cancer cell line derived from BALB/c mouse breast tissue. 4T1 cells are of epithelial origin. 4T1 cells are used to study the metastatic nature of breast cancer. In addition, they are characterized by low immunogenicity in mice, which is consistent with the characteristics of human breast adenocarcinoma. 

### 2.6. Cell Cultures

The TNBC-MDA-MB-468 cell culture and the 4T1 tumor epithelial cell line were purchased from the American Type Culture Collection (ATCC) (Biological Resources, Private Non-Profit Global Center, and Standards Organization).

Breast cancer cell line MDA-MB-468 was cultured in DMEM (Gibco) supplemented with 10% fetal bovine serum (FBS, Gibco), 1% penicillin (100 units/mL), 1% streptomycin (100 μg/mL), and 1% amphotericin B (0.25 µg/mL) (Gibco). Mouse mammary cell line 4T1 was cultured in RPMI-1640 supplemented with 10% fetal bovine serum, 1% penicillin, 1% streptomycin, and 1% amphotericin B (0.25 µg/mL) (Gibco). All cell lines were maintained at 37 °C with 5% CO_2_.

### 2.7. MTT Assay

The cytotoxic potential of the nanoparticles was determined by the MTT (3-[4,5-dimethylthiazol-2-yl]-2,5-diphenyltetrazoliumbromide) assay based on the Mosmann protocol [55,56]. 4T1 cells and MDA-MB-468 cells were seeded in DMEM culture media, separately, at a density of 1.0 × 10^4^ cells per well in 96-well plates (100 μL per well) and incubated for 24 h. After this, 10 μL or 100 μL of different test substances were added on top of the existing medium: nanoparticles suspended in deionized water, DOX solutions in DW, or nanosystems loaded with DOX in DW. For the control, only DMEM culture medium was added to the cells. The plates were maintained in an incubator in a humidified atmosphere with 5% CO_2_ at 37 °C for 0.5 h, 24 h, and 48 h. After each exposure time, the treatments contained in the plates were discarded and washed, and a solution containing MTT was added. After 4 h of incubation, a solution of dimethylsulfoxide (DMSO) was added to the wells, and the absorbance of each sample was measured using a microplate ELISA reader (Optic Ivymen System, Model 2100C, Biotech SL, Madrid, Spain) at 570 nm. Cell viability was determined by calculating the ratio of the optical density (OD) of the exposed cells to the OD of untreated cells. The mean absorbance values from eight wells were averaged for each concentration analyzed. All experiments were performed in duplicate. 

Invitrogen™ Countess II (Thermo Fisher Scientific, Waltham, MA, USA) was used to obtain the concentration required for cell research (1.0 × 10^4^ cells/well).

### 2.8. Materials’ Characterization

Determination of the hydrodynamic diameters, including the zeta potential, was carried out by dynamic light scattering (DLS) and electrophoretic light scattering (ELS) methods using a Litesizer 500 particle analyzer (Anton Paar, Graz, Austria). The pH range was from 2 to 12. A semiconductor laser served as a light source (40 MW, 658 nm).

X-ray diffraction (XRD) was used to analyze the crystal structure and phase composition of powder samples. The aqueous suspensions containing BIONs, CA-SPIONs, and DOX-CA-SPIONs were dried using a vacuum evaporator at room temperature. The obtained powders were measured using a DRON 3M X-ray diffractometer operating at a voltage of 35 kV and a current of 15 mA, using Cu Kα radiation (λ = 1.54178 Å) filtered by a nickel foil. The scanning speed was 2 degrees/min.

Transmission electron microscopy (TEM) was carried out using a JEOL 3010 transmission electron microscope equipped with a LaB6 electron gun, operating at 300 kV. The samples were prepared by drop-drying suspensions on holey carbon-foil-coated copper grids.

The magnetization curves of the synthesized magnetic nanofluids containing bare and citric-acid-modified iron oxide nanoparticles were measured on a vibrating sample magnetometer (VSM) (7300 Series VSM System, Lake Shore Cryotronics, Inc., Westerville, OH, USA) at room temperature under an applied field of up to 1.5 Tesla.

FTIR spectroscopic studies were performed on a Thermo Scientific™ Nicolet™ iS™ 5 instrument (Thermo Fisher Scientific, Waltham, MA, USA) with a diamond ATR crystal (spectral range: 4000–400 cm^−1^, 32 scans, resolution 4 cm^−1^). The colloidal suspensions were dried using a vacuum evaporator at room temperature. The measurements were performed in ATR mode at room temperature in air.

UV-visible absorption measurements of the aqueous solutions of doxorubicin and samples S1, S2, and S3 were recorded on an AVAspec HS 2048XL spectrometer (Avantes, Apeldoorn, The Netherlands) in the wavelength range of 200–1100 nm at room temperature.

## 3. Results and Discussion

### 3.1. ELS and DLS Results

**Figure 1:**
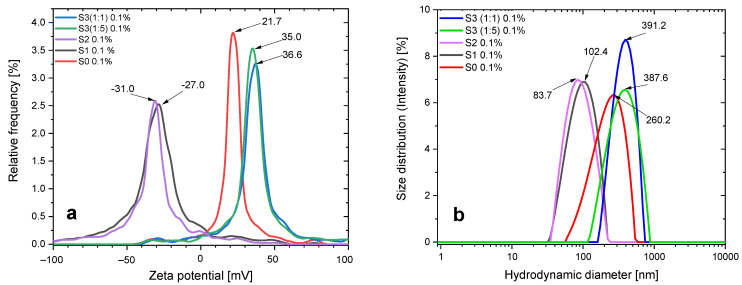
(**a**) Zeta potential and (**b**) size distribution of particle agglomerates for neutral pH (6.5) at concentrations of 0.1% *w*/*v*. S0: bare iron oxide nanoparticles; S1: CA-modified iron oxide nanoparticles (IONPs); S2: CA-modified and electrohydraulic discharge-processed IONPs; S3: CA-modified and DOX-loaded IONPs.

The zeta potential provides important information on the stability of colloidal solutions. This parameter is related to the surface charge of the particles and is an effective indicator of colloidal stability. The zeta potential values for neutral pH (6.5) (at 20 °C) for the S0, S1, S2, and S3 samples are 21.04 mV, −27.66 mV, −31.51 mV, 36.76 mV, and 34.71 mV, respectively, as shown in Figure 1.

The zeta potential of the aqueous magnetic nanosuspension containing bare iron oxide nanoparticles is positive [46,49]. This value of the zeta potential ensures the preservation of the colloidal stability of the nanofluid (for certain time) at a pH value close to physiological.

The chemisorption of CA on the surface of iron oxide nanoparticles leads to a negative surface charge with a high zeta potential (−27 mV), which indicates that CA molecules are on the surface of magnetite NPs. In contrast to bare magnetite nanoparticles, which have a positive zeta potential (21 mV), the appearance of such a high and opposite zeta potential indicates the complete coverage of magnetite nanoparticles with citric acid molecules. As for the S2 sample, it can be seen that the EHD treatment promotes deprotonation, thereby slightly increasing the magnitude of the negative zeta potential. At the same time, the surface charges of DOX-loaded S3 (1:1) and S3 (1:5) are 36.76 mV and 34.71 mV, respectively. This can be explained by the fact that cationic DOX molecules electrostatically bonded to the surface of CA-SPION diminish the negative surface charge caused by anionic carboxylate groups on their surface, and due to a large number of doxorubicin molecules, the particles acquire a positive zeta potential.

The particle size distribution (at physiological pH) was determined by the laser diffraction method with multiple scattering techniques to be in the range of roughly 90 to 380 nm. In particular, a nanofluid sample S0 shows the presence of agglomerated particles with a hydrodynamic size of up to 227 nm. Additionally, the distribution of nanoparticles in the dispersion medium is comparatively more uniform for samples treated with citric acid, S1 and S2, with hydrodynamic diameters of roughly 94 and 120 nm, respectively. The hydrodynamic size of the DOX-loaded samples (S3 (1:1) and S3 (1:5)) is up to 380 nm. The results are shown in Figure 1b and Table 1.

### 3.2. Colloidal Stability

For biological applications, the stability of nanoparticles in aqueous solutions as well as in various biological media is also crucial.

In addition to a general characterization of the hydrodynamic dimensions and zeta potential of the prepared samples, we also studied the stability of some samples both in aqueous solutions and in aqueous NaCl solutions. Intravenous infusion was chosen to mimic physiological conditions.

**Figure 2:**
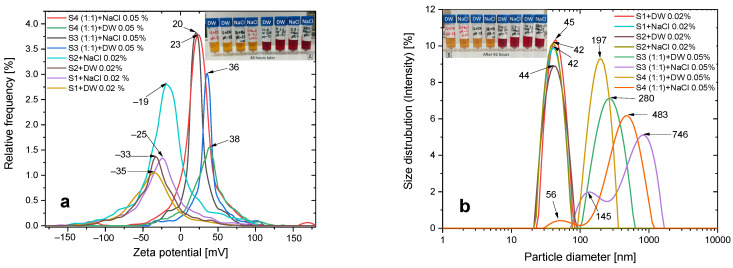
(**a**) Zeta potential and (**b**) size distribution of particle agglomerates in deionized water and sodium chloride dispersion medium for pH 7–7.1 at 20 °C. Insets show photographs of the different nanoparticle solutions (**A**) after 48 h, and (**B**) after 92 h.

The results of colloidal stability measurements are presented in Figure 2. As can be seen from the experimental data, the addition of sodium chloride solution reduces the absolute value of the zeta potential of the magnetic nanosuspension. In particular, for S3 + DW and S4 + DW samples, the zeta potential in deionized aqueous dispersion medium decreases from +38 mV and +36 mV to +23 and +20 mV for S3 + NaCl and S4 + NaCl samples, respectively (Figure 2 and Table 2). However, these values of zeta potential still ensure the sedimentation stability, which can be seen visually, as the nanofluid does not precipitate for more than 48 h. It should be noted that the change in zeta potential for these samples also leads to a change in their dimensions, in particular, the hydrodynamic dimensions of the nanoparticles in sodium chloride medium increase from 197 nm and 280 nm to 483 and 550 nm, respectively.

Similar to the behavior of doxorubicin-loaded nanocarriers, the absolute value of the zeta potential of S1 + DW and S2 + DW samples also decreases from −33 mV and −35 mV to −25 mV and −19 mV after the addition of sodium chloride, respectively. However, in contrast to the case of doxorubicin-loaded nanocarriers, the dispersion of CA-modified SPIONs in sodium chloride medium does not show significant aggregation, which is further confirmed by DLS data.

### 3.3. XRD

**Figure 3:**
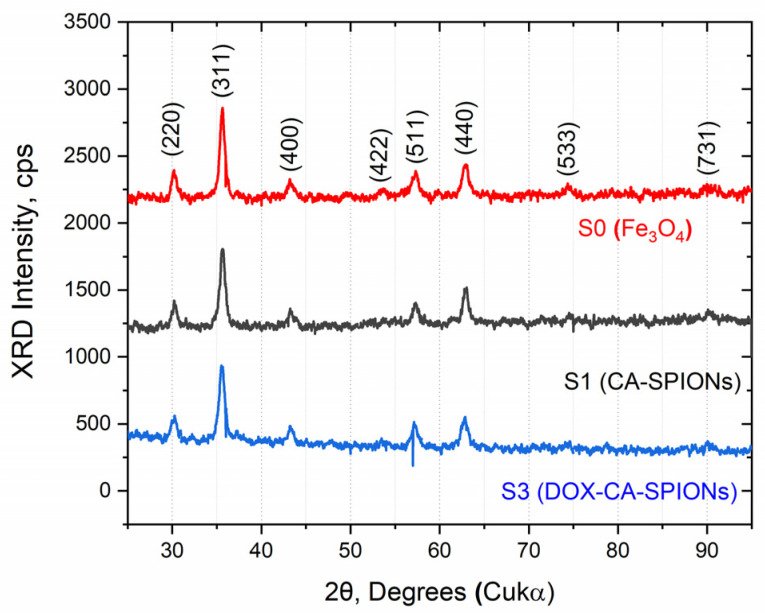
Experimental XRD patterns of BIONs, CA-SPIONs, and DOX-CA-SPIONs.

The X-ray diffraction patterns of the synthesized samples (BIONs, CA-SPIONs, DOX-CA-SPIONs) are shown in Figure 3. All diffraction patterns show the same cubic inverse spinel structure with five pronounced peaks, appearing at 2ϴ angles of 30.09°, 35.42°, 43.05°, 56.94°, and 62.51°, which correspond to the (200), (311), (400), (511), and (440) lattice reflections. The measured diffraction angles of all samples are consistent with those from the standard XRD pattern of magnetite (Fe_3_O_4_, PDF Card No. 11–614). It is noteworthy that the coating of iron oxide nanoparticles with citric acid and the subsequent loading with the anticancer drug doxorubicin do not lead to any phase change for magnetite.

The average crystallite sizes calculated using Scherrer’s equation are in the range of 22 ± 4 nm.

### 3.4. Morphology and Particle Size

**Figure 4:**
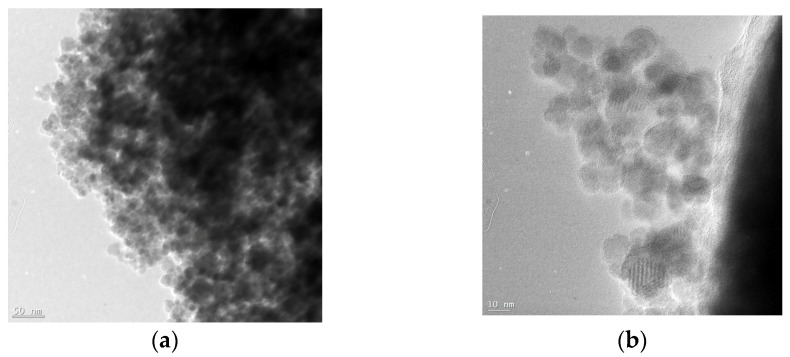
TEM images of CA-modified iron oxide nanoparticles at different magnifications (scalebars are 50 nm (**a**) and 10 nm (**b**)).

The CA-modified iron oxide nanoparticles were characterized using TEM to observe the shape and size of the nanoparticles. The iron oxide cores exhibit somewhat irregular shapes, varying from spherical to oval (Figure 4). The average size is between 15 and 18 nm, which is in good agreement with the single-particle or crystallite size obtained from the XRD data.

### 3.5. Magnetization

**Figure 5:**
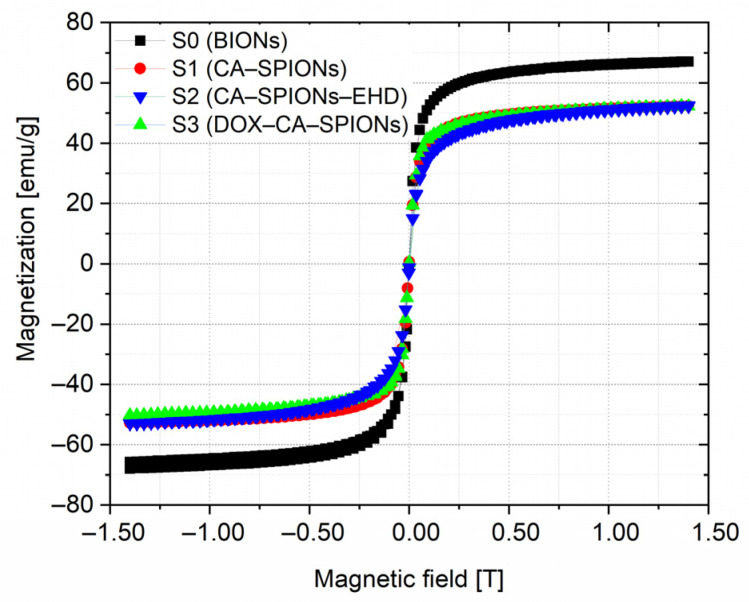
Magnetization curves of uncoated (bare) SPIONs, coated with citric acid (CA-SPION and CA-SPION-EHD), and doxorubicin-loaded iron oxide nanoparticles (DOX-CA-SPION) at 300 K.

The magnetization curves of the room-temperature VSM measurements for samples S0, S1, S2, and S3 are shown in Figure 5.

As can be seen from the magnetization curves, no hysteresis loop was detected, which indicates that the nanofluid contains single-domain nanoparticles that exhibit superparamagnetism at room temperature. The transition to saturated magnetization begins at fields of ≈0.5 Tesla. The saturation magnetization (Ms) values of BION, CA-SPION, and DOX-CA-SPION are 65.0, 52.2, and 50.4 emu/g, respectively. This outcome demonstrates that the magnetic behavior of nanoparticles is not strongly changed by the citric acid coating or the subsequent loading with the anticancer drug DOX.

### 3.6. FTIR Analysis

**Figure 6:**
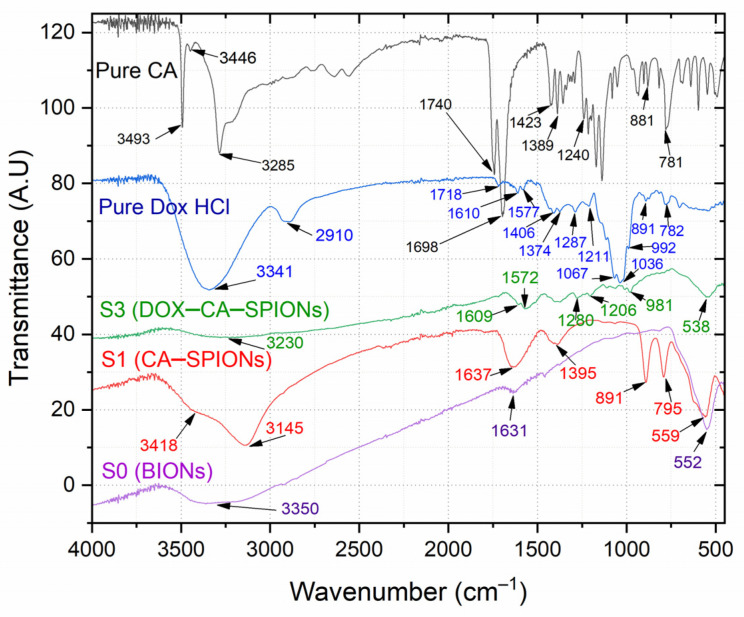
FTIR spectra of BIONs, CA-SPIONs, DOX-CA-SPIONs, magnetite, and citric acid.

The FTIR spectra of the samples S0, S1 and S3 as well as pure doxorubicin hydrochloride (Pure DOX-HCl) and pure citric acid (Pure CA) are shown in Figure 6.

In the FTIR spectrum of the uncoated particles (bare Fe_3_O_4_), a characteristic peak of magnetite is observed in the range of 550–630 cm^−1^; in particular, a peak at 552 cm^−1^ indicates the presence of magnetite. In this range, the absorption band is due to vibrations of the Fe–O bonds in the tetrahedral and octahedral positions. The vibrational bands at 3432 and 1630 cm^−1^ are due to OH groups on the surface of magnetite nanoparticles and water molecules [21].

As can be seen from the CA-SPION FTIR spectra, along with the characteristic FTIR bands of the bare magnetite, the most intense peaks correspond to the spectrum of pure citric acid (Pure CA).

In particular, the intense band at 3493 cm^−1^ and 3285 cm^−1^ in the FTIR spectrum of pure CA corresponding to the non-dissociated OH groups of CA and adsorbed water, in the FTIR spectrum of CA-SPIONs, is shifted to the band with positions of 3418 and 3145 cm^−1^, respectively [57,58].

Additionally, in the FTIR spectrum of pure CA, in the 1785–500 cm^−1^ range, the pronounced peaks at 1740 cm^−1^ and 1698 cm^−1^ correspond to symmetric and asymmetric stretching vibrations of the C=O bond in the COOH carboxyl groups of citric acid. In the FTIR spectrum of CA-SPION, this interval is shifted to 1725–1496 cm^−1^ and appears as a broad peak centered at 1637 cm^−1^, which indicates that CA molecules bind to the surface of iron oxide nanoparticles by chemisorption and a citrate ion is formed. Indeed, the carboxylate groups of citric acid form a complex with Fe atoms on the surface of magnetite nanoparticles, causing a weakening of the C=O bond in the CA molecule and a shift in the absorption band toward low energies [57,58,59,60].

However, in the FTIR spectrum of iron oxide nanoparticles coated with citric acid and loaded with DOX (DOX-CA-SPIONs), some of the aforementioned peaks characteristic of pure DOX-HCl completely disappear: 2894 cm^−1^ (C-H), 1406 cm^−1^ (C-C), 1067 cm^−1^ (C-O), and 891 and 782 cm^−1^ (N-H). For most of the other peaks [60], a decrease in intensity was observed, which is apparently associated with the electrostatic interaction of doxorubicin molecules (with a positive charge) with CA-SPIONs nanoparticles (with a negative surface potential). The above observations additionally confirm the successful loading of doxorubicin onto the iron oxide nanoparticles modified with citric acid.

### 3.7. UV-Vis Optical Studies of Anticancer Drug Loading

**Figure 7:**
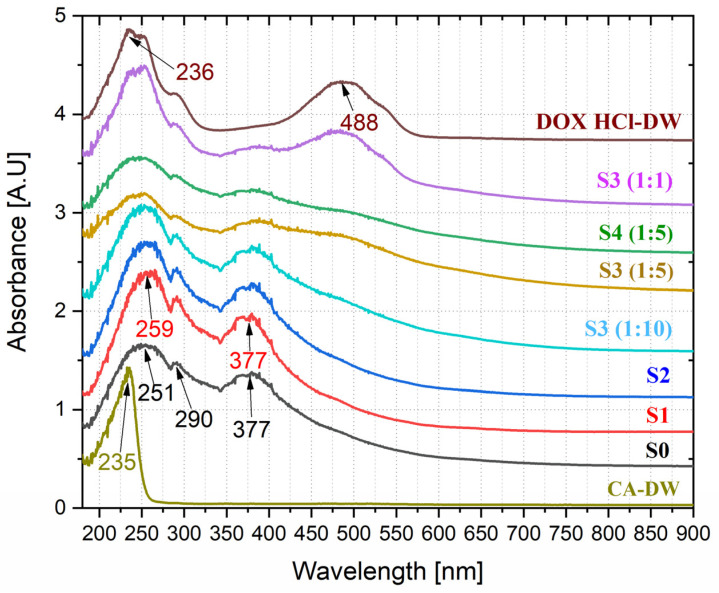
UV-VIS absorption spectra of colloidal solutions of CA, S0 (BIONs), S1 (CA-SPIONs), S2 (CA-SPIONs -EHD), S3 (DOX-CA-SPIONs (1:1), (1:5), (1:10)), and S4 (DOX-CA-SPIONS-EHD (5:1)). Concentrations were 0.05 *w*/*v* and solvent was DW.

The optical absorption spectra of magnetic nanofluids (S0, S1, S2, S3 (1:1), S3 (1:5), and S3 (1:10)), citric acid solution in DW (CA-DW), and DOX HCl solution in DW (DOX HCl-DW) are shown in Figure 7.

The absorption spectrum of the aqueous colloid of bare magnetite shows two strong absorption bands with maxima at 251 nm and 377 nm, as well as a low-intensity signal at 290 nm, while the aqueous solution of pure CA has one sharp characteristic band with a maximum at 235 nm. At the same time, we note that the absorption spectrum of the aqueous solution of pure doxorubicin has two pronounced broad signals with maxima at 245 nm and 488 nm.

For samples modified with citric acid (S1 and S2), the modification causes a slight shift in the absorption maximum from 251 nm up to 259 nm, as well as a change in the slope of the absorption curve in the extreme ultraviolet range. Additionally, it should be noted that the loading of CA-magnetic nanofluids with doxorubicin results in a DOX-specific absorption band in the 450–600 nm range.

All these results once again give a reasonable suggestion that the loading of DOX onto the nanoparticles is successful.

**Figure 8:**
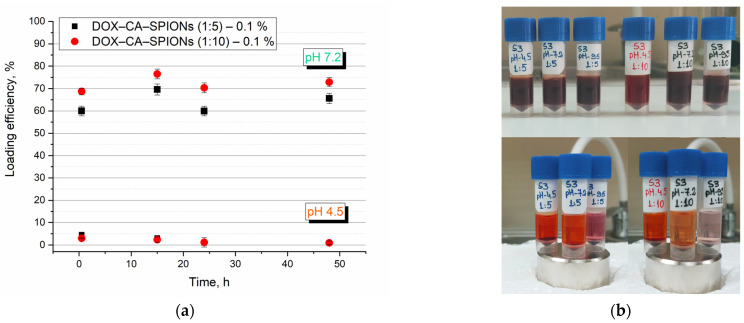
(**a**) DOX release kinetics from loaded CA-SPIONs over time at physiological pH (pH 7.2) and at acidic pH (pH 4.5) at room temperature. (**b**) Aqueous magnetic nanofluids containing iron oxide nanoparticles coated with citric acid and loaded with an anticancer drug (DOX) in a dispersion medium with a pH of 7.2, without the action of a magnetic field (top image) (30 min after sample preparation); the behavior of the same sample at different pH values of the dispersion medium, interacting with a permanent magnet (bottom image). The ratio of iron oxide nanoparticles (IONP) and anticancer drug (DOX) is as follows: DOX:IONP = 1:1; 1:5; 1:10.

We also observed the dependence of the adsorption of doxorubicin on the surface of iron oxide nanoparticles stabilized with citric acid on the pH value of the aqueous dispersion (Figure 8b).

To study the kinetics of antitumor drug loading, we placed the prepared samples on the neodymium magnet at fixed time intervals, and kept on it for 40 min. From the supernatant of the precipitated nanodispersion, using a UV-visible spectrophotometer, the optical density of the remaining DOX in the supernatant was measured at 480 nm (except for the 48 h measurement, these samples were not in contact with the magnet and were decanted with a permanent neodymium magnet immediately prior to measurement).

The absorbance of the supernatant at 480 nm was used to quantify the loading efficiency (LE) of DOX, using the equation:

(1) $$LE\left(\%\right)=\frac{{ABS}_{TDOX}-{ABS}_{FDOX}}{{ABS}_{TDOX}}\times 100$$

where *ABS_TDOX_* is the absorption intensity value of the drug fed and *ABS_FDOX_* is the absorption intensity value of free (nonloaded on the surface of nanoparticles) drug in the supernatant. The corresponding graph is presented in Figure 8a.

As seen in the graph (Figure 8a), the release of doxorubicin at physiological pH values has a similar characteristic for both samples. However, as expected, its amount in the sample with a higher concentration of magnetic nanoparticles, S3 (10:1), exceeds that of S3 (5:1) and amounts to 75 and 62%, respectively. As for the loading efficiency at pH 4.5, it is not significant, although it is maximal in the first 30 min and then approaches zero. The fact that DOX is released at acidic pH suggests that this anticancer drug may be released preferentially in the cell’s endosomal or lysosomal compartment, which is shielded from drug efflux.

This indicates that the CA-SPION nanoparticles after loading with DOX are pH-sensitive to the release of the drug, which is mainly attributed to the functionalization of the surface by carboxyl groups. At acidic pH, carboxyl groups are uncharged and interact weakly with NH_3_^+^ in DOX. However, as the pH increases, the carboxyl groups gain a negative charge and attract the positively charged molecules, which have a stronger interaction with negatively charged CA-SPIONs, and the adsorption efficiency increases [61].

Thus, the adsorption of doxorubicin on iron nanoparticles modified with citric acid depends both on the pH value of the dispersion medium and on the concentration of nanoparticles.

### 3.8. In Vitro Cytotoxicity

#### 3.8.1. Sample Preparation

The purpose of the biological studies was to evaluate the cytotoxicity (effect on cell viability) of the anti-cancer drug doxorubicin and magnetic nanofluids containing bare (BION), citric-acid-modified (CA-SPION), and citric-acid-modified/doxorubicin-loaded (DOX-CA-SPIONs/DOX-CA-SPIONs-EHD), taken at various concentrations, on the cancer models 4T1 (mouse tumor epithelial cells) and MDA-MB-468 (human TNBC cells) using the MTT method.

In a 96-well plate, 1.0 × 10^4^ tumor cells/well were cultivated for 24 h. Afterward, different amounts (10 μL and 100 μL) of the nanosystems described above were added to the wells. Cytotoxicity and cell viability were evaluated in the initial 0.5 h, 24 h, and 48 h phases. The test was performed following the kit protocol (Sigma: CGD1-1KT) as described in Section 2.7. MTT assay. The concentrations of the samples used in the study and the corresponding abbreviations are shown in Table 3.

#### 3.8.2. 4T1 Cancer Cell Line

**Figure 9:**
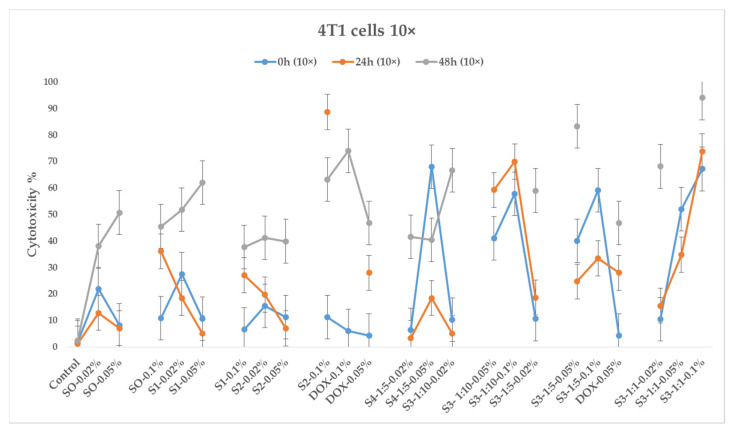
The effect of nanoparticles (10x dose, 100 μL) on the 4T1 cell line, where the control is untreated tumor cells; DOX is present at concentrations of 0.05%, 0.1% *w*/*v*; S0—BIONs (0.02%, 0.05% and 0.1% *w*/*v*); S1—SPIONs modified with citric acid; S2—EHD-processed CA-SPIONs; S3—CA-SPIONs loaded with DOX (DOX/SPIONs = 1:10, 1:5, 1:1) with concentrations of 0.02%, 0.05%, and 0.1% *w*/*v*; S4—DOX-loaded, EHD-processed CA-SPIONs (1:5) with concentrations of 0.02% and 0.05% *w*/*v* (MTT analysis—calculated optical density at 570 nm, *p*-value < 0.05).

**Figure 10:**
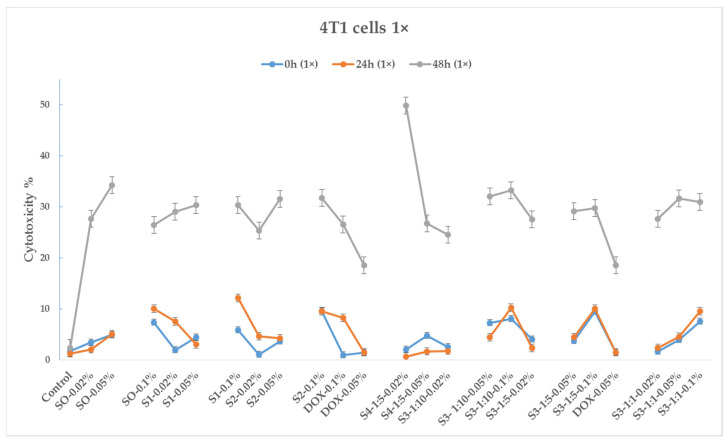
The effect of nanoparticles (1× dose, 10 μL) on the 4T1 cell line, where the control is untreated tumor cells; DOX is represented by concentrations of 0.05%, 0.1% *w*/*v*; S0—BIONs (0.02%, 0.05% and 0.1% *w*/*v*); S1—SPIONs modified with citric acid; S2—EHD-processed CA-SPIONs; S3—DOX-loaded CA-SPIONs (DOX/SPIONs = 1:10, 1:5, 1:1) with concentrations of 0.02%, 0.05%, and 0.1%; S4—DOX-loaded, EHD-processed CA-SPIONs (1:5) with concentrations of 0.02% and 0.1% *v/w* (*p*-value < 0.05).

Figure 9 and Figure 10 depict 30 min, 24 h, and 48 h cytotoxicity charts on the 4T1 cancer cell line for the samples with 10× and 1× concentrations (100 μL and 10 μL, respectively).

As seen from the diagrams, bare iron oxide nanoparticles (sample S0) exhibit cytotoxic effects on 4T1 tumor cells, and their effect is expressed over time and increases in phases of 24 and 48 h. The cytotoxic effect depends on the amount of added test samples (dose) to the cell environment; in particular, cytotoxicity is more pronounced when cells are treated with 100 μL of the test substance.

Like bare magnetite nanoparticles, the citric-acid-coated magnetic nanoparticles (sample S1) exhibit cytotoxicity toward 4T1 tumor cells.

As for sample S2, treated with EHD, here, as in the previous samples, a cytotoxic effect on tumor cells was observed. It is noteworthy that the 0.1% variation of this sample (dose 100 μL) in the 24 h phase shows 89% cytotoxicity, while S2–0.05% and S2–0.02% samples show cytotoxicities of 7% and 20% (12 and 4.5 times less, respectively). Additionally, in the 48 h phase, the cytotoxicity of sample S2–0.1% decreases to 70%, while those of samples S2–0.02% and S2–0.05% increase to 40%. The cytotoxicity of all samples in the 10 μL case is quite similar.

The sample S3–1:1–0.1% (100 μL) exhibits the highest cytotoxicity manifested over time (70, 75, and 95% cytotoxicity, in the 30 min, 24 h, and 48 h phases, respectively). With the 10 μL dose, the cytotoxic effect is much weaker and reaches 30% only after 48 h.

The sample S4–1:5–0.05% (DOX-loaded EHD-processed CA-SPIONs), used in a dose of 100 μL, exhibits a strong cytotoxic effect (68%) in the 30 min phase; however, this effect quickly declines to 18% in the 24 h phase and increases again to 40% at 48 h. This sample is nearly as cytotoxic as DOX–0.05%, demonstrating the synergic nature of this combination. As for the S4–1:5–0.02% variant, the 30 min phase does not show high cytotoxicity, but after 48 h, the cytotoxicity increases to more than 40%.

Of the four tested CA-SPIONs, S3 is of particular interest. The sample with the highest cytotoxicity value in the 24 h phase is S3–1:1–0.1% (75% cytotoxicity). It should be emphasized that this sample contains the same amounts of doxorubicin and CA-SPION as DOX–0.05% and S1–0.05%, respectively. The cytotoxicity of sample S1–0.05% is 14 times lower than that of sample S3–1:1–0.1% on its own. Additionally, it is important to note that sample DOX–0.05% of pure doxorubicin has a 28% individual cytotoxicity rating, which is 2.6 times lower than that of sample S3–1:1–0.1%. Thus, in this case, there is an obvious synergistic effect. A weaker synergistic impact is also observed in the S3–1:1–0.05% variation, but it is also important to note that the amount of the doxorubicin and CA-SPIONs in the S3–1:1–0.02% variation is 4 times less in comparison with samples DOX–0.05% and S1–0.05%.

The samples S3–1:10–0.1%, S3–1:10–0.05%, and S3–1:5–0.1% also show a potent synergistic effect.

In summary, according to the results of an experimental study on cytotoxicity in cancer 4T1 cell lines, S3 samples (DOX-CA-SPIONs) indicate a synergistic effect, opening up significant potential for drug dose reduction. The significant cytotoxicity of the S2–0.1% *w*/*v* sample throughout the 24 h phase is also remarkable.

#### 3.8.3. MDA-MB-468 Cancer Cell Line

**Figure 11:**
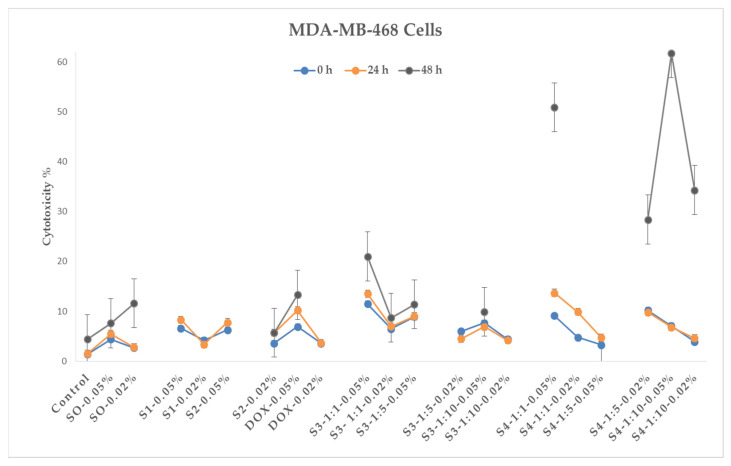
Cytotoxic effects of nanoparticles on the MDA-MB-468 cell line 1× concentration (MTT assay—estimated optical density at 570 nm). The control is untreated tumor cells. DOX is represented by concentrations of 0.02%, 0.05% *w*/*v*; S0—BIONs (0.02%, 0.05% *w*/*v*); S1—SPIONs modified with citric acid; S2—EHD-processed CA-SPIONs (0.05%, 0.02% *w*/*v*); S3—DOX-loaded CA-SPIONs (DOX/SPIONs = 1:10, 1:5, and 1:1), with concentrations of 0.02%, 0.05%; S4—DOX-loaded, EHD-processed CA-SPIONs (1:10, 1:5, and 1:1) with concentrations of 0.02% and 0.5% *w*/*v*. (*p*-value < 0.05).

The triple-negative breast tumor cell line MDA-MB-468 was treated with the same samples as the 4T1 tumor cells, although in this case, only 10 μL of test substance was added to the cell area (Figure 11). The inhibitory effect of iron oxide nanoparticles on the proliferation of tumor cells is demonstrated for this cell line as well, and the effect is also expressed over time. Sample S3 (magnetic doxorubicin nanoparticles) also shows a synergistic effect on MDA-MB-468 cell cultures compared to doxorubicin.

Separating the cytotoxic effects of sample S4, variations of S4–1:1–0.05% (cytotoxicity 52%) and S4–1:10–0.05% (cytotoxicity 62%) of this sample show a pronounced synergistic effect after the 24 h phase (compared to S2 and DOX); however, it should also be noted that in the 48 h phase, the same samples show lower magnitudes of cytotoxic effects.

The results of in vitro studies show that iron oxide nanoparticles, both bare and citric-acid-modified (including samples treated with electrohydraulic discharge), exhibit a cytotoxic effect on tumor cells. In addition, the combination of doxorubicin plus a magnetic nanosystem shows a synergistic effect, which, in our opinion, is associated with the following circumstances. Several mechanisms of the effect of nanoparticles on cells and tissues are discussed in the scientific literature. The most advanced hypothesis of nanoparticle toxicity is the formation of reactive oxygen species (ROS), which cause damage to proteins, DNA, and tissues [62]. Reactive oxygen species can form on the surface of nanoparticles directly from free radicals, or nanoparticles of transition metals such as iron can generate reactive oxygen species (namely hydroxyl radicals) that act as catalysts in the Fenton reaction [27,34,35].

At the same time, the formation of active forms of oxygen is a well-known mechanism involved in DOX-induced apoptosis in various tumor cells. The synergistic effect of DOX and magnetic nanoparticles is attributed to the enhanced penetration and cellular uptake of the drug, facilitated by the endocytosis of the charged nanoparticles [63].

**Figure 12:**
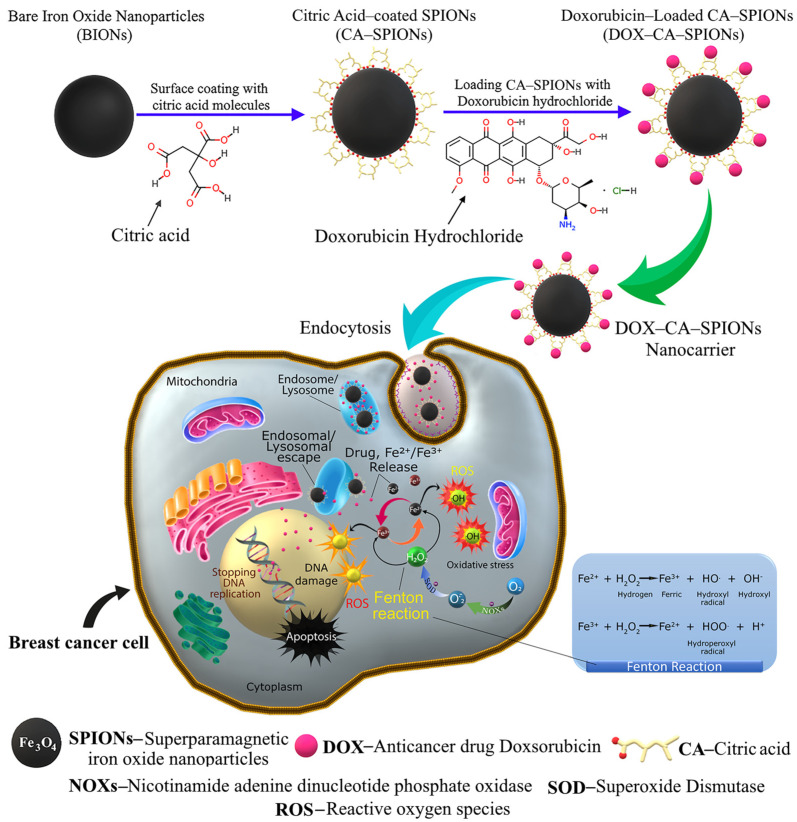
Schematic representation of the construction of the DOX-CA-SPIONs nanocarrier and the underlying anticancer mechanism. First, citric acid is coated onto SPION and DOX is loaded onto CA-SPIONs to create the pH-sensitive core–shell system (DOX-CA-SPIONs). Next, the DOX-CA-SPIONs nanocarriers are internalized into the cancer cell by endocytosis with subsequent formation of endocytic vesicles. After the release from lysosomes of an anti-cancer drug and Fe^2+^/Fe^3+^ into the intracellular milieu, iron ions undergo a Fenton-like reaction accompanied by the production of many hydroxyl radicals, thereby increasing the content of ROS, which contributes to oxidative stress. In addition, DNA synthesis is inhibited as a result of doxorubicin’s effects on the cell nucleus, and ultimately, these effects contribute to the death of cancer cells.

It should also be noted that the active forms of oxygen induced by iron oxide nanoparticles enhance the cytotoxic effect. From the aforementioned, it can be assumed that damage caused by the generation of oxygen-active forms of cell DNA is one additional potential mechanism for magnetic-nanoparticle-induced cellular apoptosis, in addition to DOX-induced cellular apoptosis (Figure 12).

## 4. Conclusions

In this study, we presented the synthesis of citric-acid-coated super-paramagnetic iron oxide nanoparticles through an automated chemical reactor by sonochemical coprecipitation. These nanoparticles were further loaded with the anti-cancer drug doxorubicin, resulting in a water-based magnetic nanofluid with excellent sedimentation stability and strong magnetic properties.

In vitro studies conducted in triple-negative breast cancer models demonstrated a synergistic effect in inhibiting the growth and proliferation of cancer cells when compared to treatment with pure doxorubicin. This synergistic effect showed the potential for utilizing the iron-oxide-nanoparticle-mediated targeted drug delivery system to optimize drug doses and reduce the side-effects of doxorubicin while enhancing its cytotoxic effect on cancer cells in tumor therapy. By utilizing the magnetic properties of the nanofluids, targeted delivery of doxorubicin to cancer cells can be achieved, increasing its efficacy and minimizing its impact on healthy cells.

The findings of this study highlight the novelty and potential of combining the drug with magnetic nanocarriers for improved therapeutic outcomes. Furthermore, application of the novel method of electrohydraulic discharge allows the control of the properties of iron oxide nanoparticles prepared by the traditional co-precipitation technique. 

However, it is important to acknowledge the limitations of this work. Further investigations are required to assess the long-term stability, biocompatibility, and targeted delivery efficiency of the developed nanofluids in complex biological systems. Additionally, in vivo studies and clinical trials are essential to validate the effectiveness and safety of this approach for cancer treatment.

## Figures and Tables

**Table 1:** ELS and DLS experimental results of samples.

Samples	Mean Zeta Potential (mV)	Electrophoretic Mobility (µm cm/Vs)	Conductivity (mS/cm)	Hydrodynamic Diameter (nm)	Peak Analysis Intensity (nm)	Polydispersity Index (%)
S0	21.0	1.63	0.007	226.4	260.2	26.2
S1	−27.7	−1.96	0.003	93.6	102.4	20.5
S2	−31.5	−2.45	0.01	93.8	86.9	22.4
S3 (1:1)	36.8	2.60	0.24	380.9	391.3	18.6
S3 (1:5)	34.7	2.46	0.90	375.6	386.2	24.2

**Table 2:** ELS and DLS experimental results.

Samples	Mean Zeta Potential (mV)	Electrophoretic Mobility (µm cm/Vs)	Conductivity (mS/cm)	Hydrodynamic Diameter (nm)	Standard Dev. (nm)	Polydispersity Index (%)
S1 + DW 0.02%	−29.92	−2.12	0.54	46.6	11.8	12.4
S1 + NaCl 0.02%	−23.29	−1.64	6.92	42.2	11.3	11.7
S2 + DW 0.02%	−33.88	−2,40	0.43	43.2	13.7	10.2
S2 + NaCl 0.02%	−23.82	−1.68	7.70	42.6	11.0	12.6
S3 (1:1) + DW 0.05%	36.56	2.59	0.11	271.0	104.1	15.8
S3 (1:1) + NaCl 0.05%	20.51	1.45	6.57	550.4	387.9	25.0
S4 (1:1) + DW 0.05%	38.36	2.72	0.04	198.1	61.6	17.9
S4 (1:1) + NaCl 0.05%	23.28	1.64	5.73	455.7	223.2	26.3

**Table 3:** The concentrations of the samples used in the study and the corresponding abbreviations.

Sample’s Designations	Solid Component	Designations Corresponding to Different Concentrations (*w*/*v*)	DOX Concentration (mM)	Concentration, (mg/mL)
DOX	Doxorubicin	DOX–0.1%	1.84	
		DOX–0.05%	0.92	
		DOX–0.02%	0.37	
S0	BIONs – Bare iron oxide nanoparticles	S0–0.1%		1.00
		S0–0.05%		0.50
		S0–0.02%		0.20
S1	CA-SPIONs – Citric acid-coated iron oxide nanoparticles	S1–0.1%		1.00
		S1–0.05%		0.50
		S1–0.02%		0.20
S2	CA-SPIONs-EHD – Electrohydraulic discharge-processed CA-SPIONs	S2–0.1%		1.00
		S2–0.05%		0.50
		S2–0.02%		0.20
S3	DOX-CA-SPIONs – Doxorubicin-loaded CA-SPIONs	S3–1:1–0.1%	0.92	0.50
		S3–1:1–0.05%	0.46	0.25
		S3–1:1–0.02%	0.18	0.10
		S3–1:5–0.10%	0.31	0.83
		S3–1:5–0.05%	0.15	0.42
		S3–1:5–0.02%	0.06	0.17
		S3–1:10–0.10%	0.17	0.91
		S3–1:10–0.05%	0.08	0.45
		S3–1:10–0.02%	0.03	0.18
S4	DOX-CA-SPIONs-EHD – Electrohydraulic discharge-processed DOX-CA-SPIONs	S4–1:1–0.1%	0.92	0.50
		S4–1:1–0.05%	0.46	0.25
		S4–1:1–0.02%	0.18	0.10
		S4–1:5–0.1%	0.31	0.83
		S4–1:5–0.05%	0.15	0.42
		S4–1:5–0.02%	0.06	0.17
		S4–1:10–0.1%	0.17	0.91
		S4–1:10–0.05%	0.08	0.45
		S4–1:10–0.02%	0.03	0.18

## Data Availability

Not applicable.
